# Novel pathogenic ATM mutation with ataxia-telangiectasia in a Chinese family

**DOI:** 10.3389/fgene.2024.1491649

**Published:** 2024-11-28

**Authors:** Qiaomin Zhou, Minling Chen, Enfu Tao

**Affiliations:** ^1^ Department of Eugenic Genetics, Wenling Maternal and Child Healthcare Hospital, Wenling, Zhejiang, China; ^2^ Department of Maternity, Wenling Maternal and Child Healthcare Hospital, Wenling, Zhejiang, China; ^3^ Department of Neonatology and NICU, Wenling Maternal and Child Healthcare Hospital, Wenling, Zhejiang, China

**Keywords:** ataxia-telangiectasia, ATM gene, frameshift mutation, cerebellar atrophy, muscle atrophy, immunodeficiency, alpha-fetoprotein

## Abstract

Ataxia-Telangiectasia (A-T) is a rare, autosomal recessive disorder characterized by progressive cerebellar ataxia, oculocutaneous telangiectasia, immunodeficiency, and increased cancer risk. Mutations in the ATM gene, which is essential for DNA damage repair, underlie this condition. This study reports a novel homozygous frameshift mutation (ATM_ex20 c.3062delT, p. Val1021fs) in a Chinese family with two affected siblings. The mutation, located in exon 20, has not been previously documented, expanding the spectrum of ATM mutations. The proband and her older sister presented with classic A-T symptoms, including gait instability and conjunctival telangiectasia. Both siblings presented with immunodeficiency, characterized by low immunoglobulin A (IgA) levels, slightly elevated IgM levels, and elevated alpha-fetoprotein (AFP). Cranial magnetic resonance imaging (MRI) findings revealed cerebellar atrophy and cerebral white matter lesions in both sisters. Interestingly, while both sisters shared the same mutation, their clinical severity differed, highlighting the complexity of genotype-phenotype correlations in A-T. The parents and an unaffected sister were heterozygous carriers, consistent with autosomal recessive inheritance. This study underscores the importance of genetic testing in A-T diagnosis and provides new insights into the genetic diversity of ATM-related diseases. Further research is needed to understand the broader implications of this mutation.

## Introduction

Ataxia-Telangiectasia (A-T), also known as Louis-Bar syndrome, is a rare, autosomal recessive disorder that affects multiple organ systems, particularly the nervous system. It is characterized by progressive cerebellar ataxia, oculocutaneous telangiectasia, immunodeficiency, and an increased risk of malignancies, especially lymphomas and leukemias (Teive et al., 2015; Aguado et al., 2022; Rothblum-Oviatt et al., 2016). The disease typically manifests in early childhood, with affected individuals often presenting with gait disturbances, slurred speech, and a range of other neurological and systemic symptoms (Shiloh, 1995; De Nardi et al., 2023).

The genetic basis of A-T lies in mutations in the Ataxia-Telangiectasia Mutated (ATM) gene, which plays a crucial role in the cellular response to DNA damage (Mitiagin and Barzilai, 2023). The ATM protein is involved in the detection and repair of double-strand breaks in DNA, and mutations in this gene lead to the accumulation of genetic damage, contributing to the disease’s clinical manifestations (Lee, 2024). To date, over 3,000 distinct disease-related ATM mutations have been identified in patients with A-T. With significant variability in clinical presentation among patients (Amirifar et al., 2020). This variability poses challenges in establishing clear genotype-phenotype correlations, complicating both diagnosis and genetic counseling (Cao et al., 2019).

In this study, we report the identification of a novel ATM gene mutation in a Chinese family with two affected siblings. This mutation, located at exon 20 of the ATM gene (c.3062del, p. Val1021fs), has not been previously documented in scientific literature. By investigating this specific case, we aim to expand the known spectrum of ATM gene mutations and shed light on the clinical implications of this new mutation. The findings underscore the importance of genetic testing and family studies in diagnosing and managing A-T, particularly in populations where specific mutations may be underreported (Rothblum-Oviatt et al., 2016; Amirifar et al., 2019).

## Case description

**FIGURE 1:**
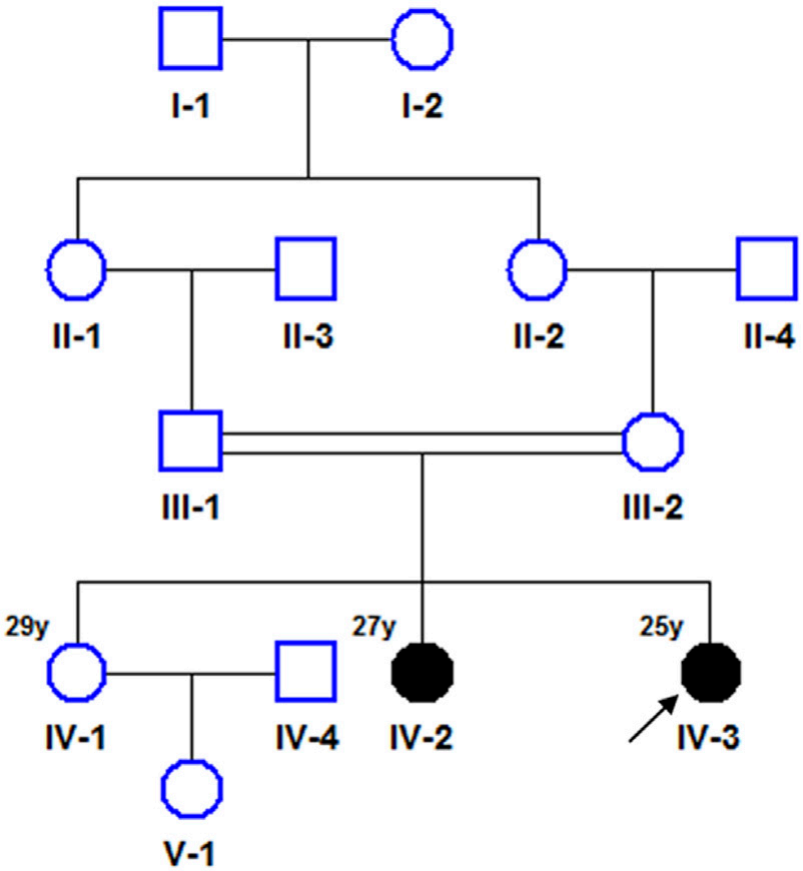
Pedigree of the family with Ataxia-Telangiectasia. Squares represent males, circles represent females, filled symbols denote affected individuals, and arrows indicate the probands.

**FIGURE 2:**
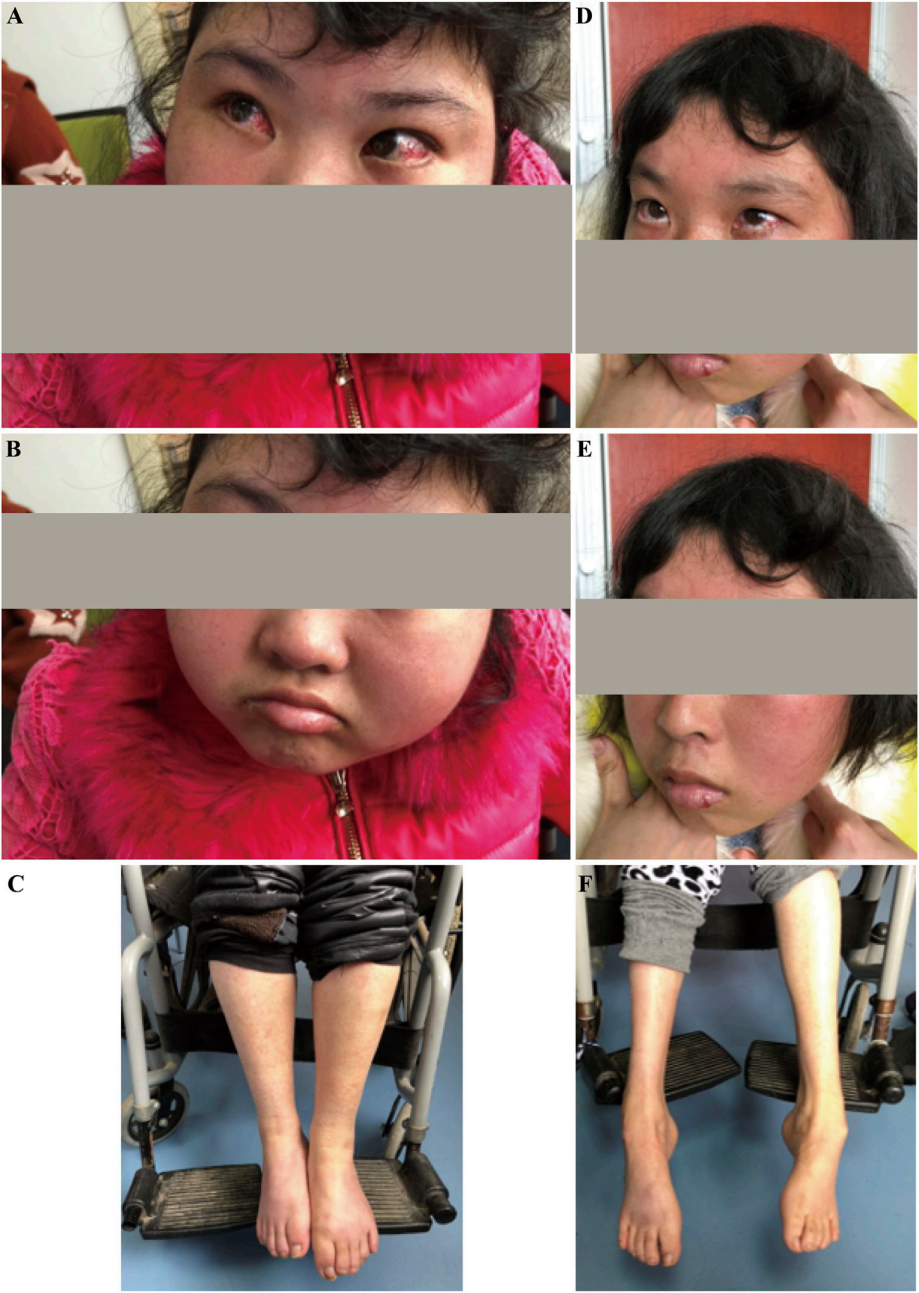
Clinical presentation of the affected sisters with Ataxia-Telangiectasia. **(A–C)**: The younger sister exhibited conjunctival congestion, facial flushing, intellectual disability, reliance on a wheelchair, and no muscle atrophy. **(D–F)**: The older sister presented with conjunctival congestion, facial flushing, intellectual disability, reliance on a wheelchair, and muscle atrophy.

A proband (IV-3; age 25) and her elder sisters (IV-2, age 27, and IV-1, age 29), along with their parents (III-1 and III-2), were referred to our clinic from a Han Chinese family in eastern China due to developmental regression observed in the two younger sisters. The parents of the sisters are first cousins, indicating a consanguineous marriage, which increases the likelihood of autosomal recessive inheritance. The pedigree is shown in Figure 1. Like her older sister, the proband exhibited delayed motor and speech development, with symptoms progressively worsening since childhood. At the time of reporting, she retained some ability to walk with assistance but displayed evident gait instability and dysarthria. Physical examination revealed conjunctival telangiectasia and facial capillary dilation, similar to her older sister (Figures 2A, B). However, unlike her sister, she did not experience significant muscle atrophy (Figure 2C). Her older sister (IV-2, 27 years old) also presented with delayed motor milestones, including a late onset of walking and difficulty maintaining balance. By the ages of 7 to 8, her condition worsened, marked by pronounced gait instability and dysarthria. By 15 to 16, she became unable to walk independently and required support from walls or other aids. Physical examination showed conjunctival telangiectasia, facial erythema due to dilated capillaries, and mild cognitive delay (Figures 2D, E). Additionally, she exhibited progressive muscle atrophy, particularly in the lower limbs, along with bilateral foot deformities (inward turning of the feet) (Figure 2F). Despite her physical challenges, she remains socially active and displays a cheerful personality. The clinical presentation of the two affected sisters were shown in Figure 2. No abnormalities were observed in her oldest sister (IV-1, 29 years old) and their parents (III-1 and III-2). A familial genetic disorder was suspected, prompting the decision to perform whole exome sequencing to identify potential genetic abnormalities.

**FIGURE 3:**
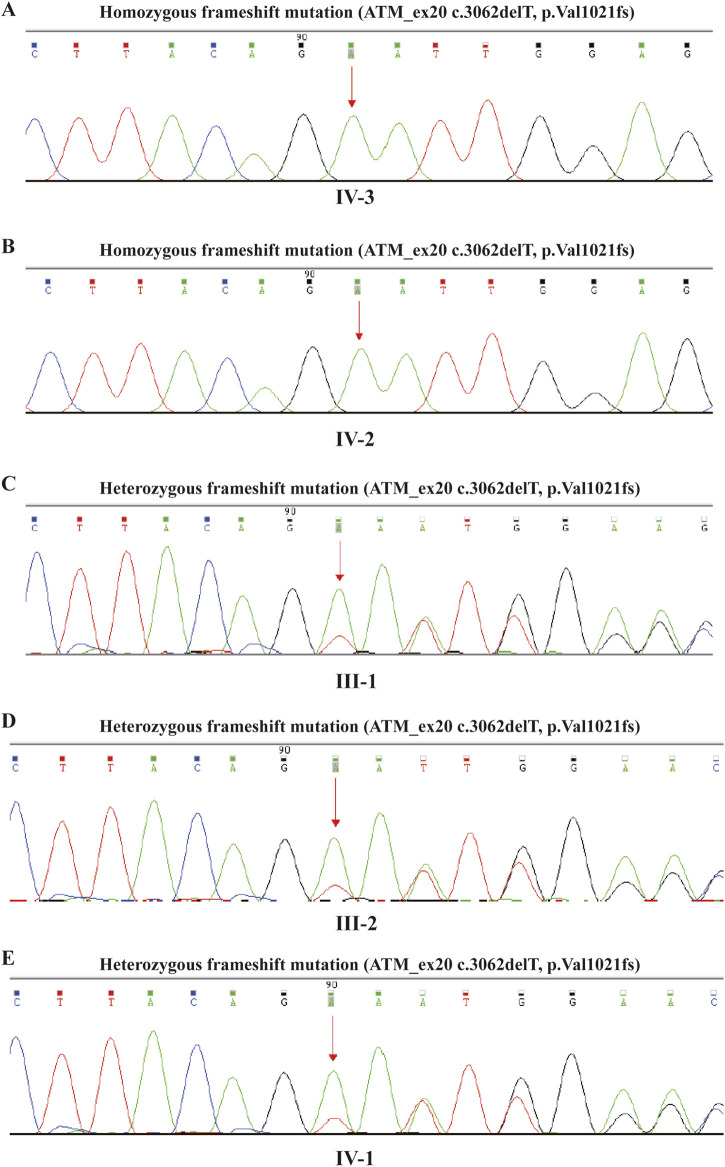
Novel homozygous and heterozygous frameshift mutations in the ATM Gene of family. **(A, B)**: homozygous frameshift mutation (ATM_ex20 c.3062delT, p. Val1021fs) identified in the two affected sisters. **(C–E)**: heterozygous frameshift mutation (ATM_ex20 c.3062delT, p. Val1021fs) found in their parents and the eldest sister respectively.

A pathogenic mutation was detected: ATM_ex20 NM_000051.3, c.3062delT (p.Val1021fs). This frameshift mutation is in a homozygous state and follows an autosomal recessive inheritance pattern. It has been classified as pathogenic and is associated with A-T. This specific mutation has not been previously reported in scientific literature, but frameshift mutations can lead to truncated proteins or protein degradation, significantly impacting protein function and potentially causing disease. We conducted high-throughput sequencing and analysis of the coding regions of genes related to hereditary diseases, focusing on 1,170 genes associated with neurological disorders. The proband carries a homozygous pathogenic frameshift mutation (ATM_ex20 c.3062delT, p. Val1021fs). Although not previously reported in the literature, this mutation is likely to result in a truncated or degraded protein, potentially causing disease. Family testing confirmed that the proband and her older sister carry this mutation in a homozygous state, while their parents and oldest sister are heterozygous carriers. Given the clinical presentation and the family segregation pattern, this mutation is classified as pathogenic. The genetic condition related to the ATM gene follows an autosomal recessive inheritance pattern, consistent with the clinical diagnosis of A-T. Sanger sequencing was used to validate the candidate variants after data analysis. The sanger sequencing results for the family are shown in Figure 3. The proband (Figure 3A) and her older sister (Figure 3B) were both found to carry a homozygous pathogenic frameshift mutation (ATM_ex20 c.3062delT, p. Val1021fs). In contrast, her oldest sister (Figure 3E) and parents (Figures 3C, D) were identified as heterozygous carriers of the same frameshift mutation (ATM_ex20 c.3062delT, p. Val1021fs). Accordingly, a novel pathogenic mutation in the ATM gene linked to A-T was confirmed.

**FIGURE 4:**
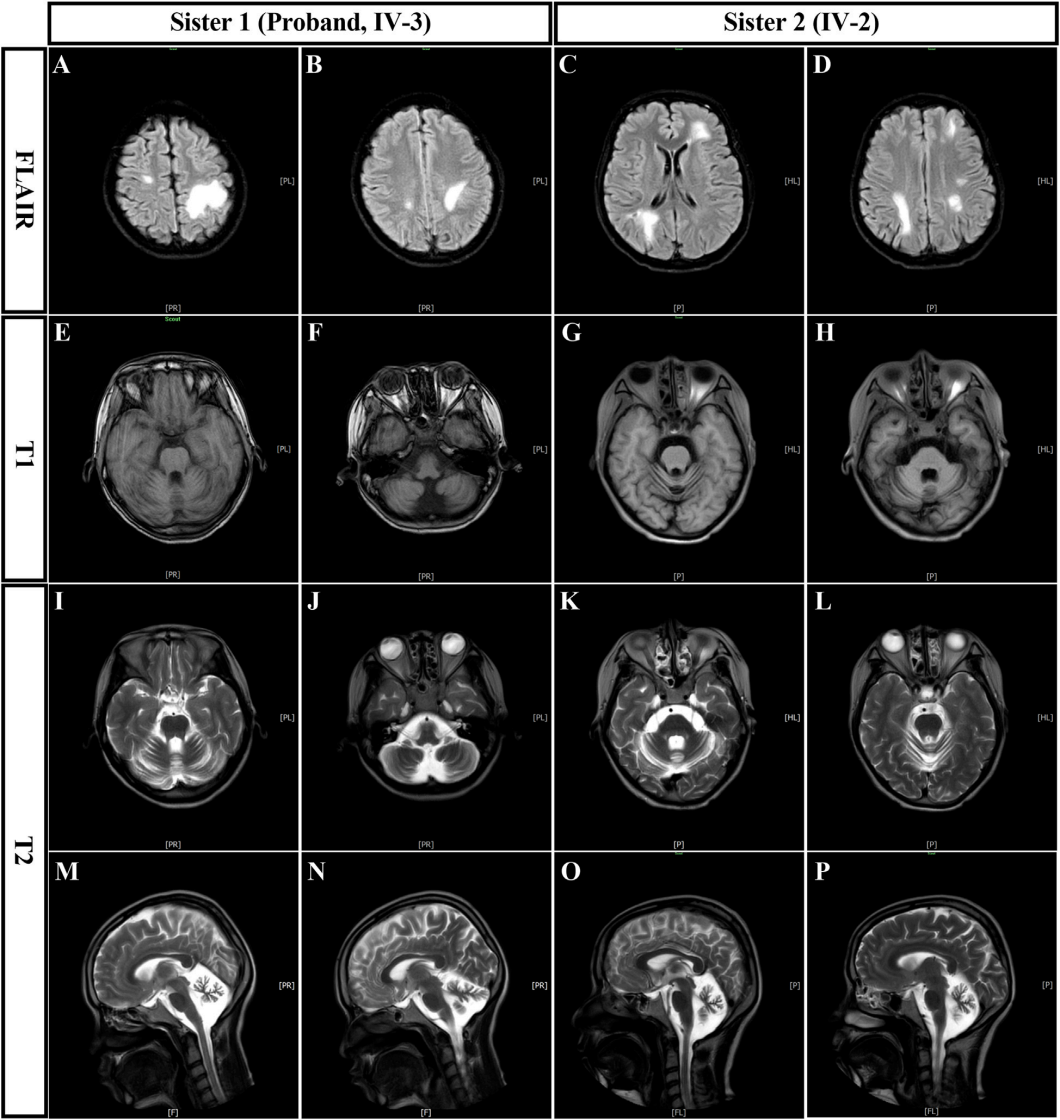
Representative cranial MRI of the affected sisters with Ataxia-Telangiectasia. **(A, B)**: The FLAIR sequences for Sister 1 (IV-3) displayed patchy high-signal lesions in the right frontotemporal lobe and left parietal lobe, suggesting white matter abnormalities. **(C, D)**: The FLAIR sequences for Sister 2 (IV-2) also demonstrated similar patchy high-signal lesions in the left frontal lobe and bilateral parietal lobes. **(E, F)**: The T1-weighted images for Sister 1 revealed mildly low signals, with no obvious abnormalities in the brainstem or cerebellum. **(G, H)**: The T1-weighted images for Sister 2 showed an isointense signal. **(I, J)**: The T2-weighted axial images for Sister 1 showed a high signal. **(K, L)**: The T2-weighted axial images for Sister 2 demonstrated a high signal. **(M, N)**: The sagittal T2-weighted images for Sister 1 showed deepened cerebellar sulci, indicating cerebellar atrophy. **(O, P)**: The sagittal T2-weighted images for Sister 2 revealed similar findings of cerebellar atrophy, confirming structural degeneration. MRI, cranial magnetic resonance imaging.

The proband was followed up for 5 years and passed away at the age of 30 due to a pulmonary infection and malignancy. Three months before her death, an immunological evaluation revealed immunoglobulin A (IgA) < 0.12 g/L (reference range: 1.0–4.2 g/L), elevated IgM at 4.15 g/L (reference range: 0.5–2.8 g/L), and increased IgG at 22.28 g/L (reference range: 8.6–17.40 g/L). Complement analysis showed elevated C3 at 1.509 g/L (reference range: 0.70–1.40 g/L) and C4 at 0.374 g/L (reference range: 0.1–0.4 g/L), indicating immune dysregulation. T cell subsets revealed total T lymphocytes at 58.39% (reference range: 50.00%–84.00%), helper T cells (CD3^+^ CD4^+^) at 40.29% (reference range: 27%–51%), and cytotoxic T cells (CD3^+^ CD8^+^) at 15% (reference range: 15%–44%), with a CD4^+^/CD8^+^ ratio of 2.69 (reference range: 0.71–2.87). Additionally, her alphafetoprotein (AFP) level was markedly elevated at 9,166.17 ng/mL (reference range: ≤7.329 ng/mL), suggesting significant abnormality. Cranial magnetic resonance imaging (MRI) showed cerebellar atrophy and cerebral white matter lesions in the right frontotemporal lobe and left parietal lobe (Figure 4). In addition, her older sister 2 (IV-2), to date, has not been diagnosed with any malignancies. However, her mobility has progressively declined, and she is now unable to walk independently, relying on walls or other aids for support. During her most recent follow-up at the age of 32, her Scale for the Assessment and Rating of Ataxia (SARA) (Schmitz-Hübsch et al., 2006; Perez-Lloret et al., 2021) score was 30, with individual components as follows: Gait 7, Stance 6, Sitting 2, Speech Disturbance 3, Finger Chase 3, Nose-Finger Test 3, Fast Alternating Hand Movements 3, and Heel-Shin Slide 3. Her AFP level was significantly increased at 318.59 ng/mL. Immunological evaluation revealed low IgA at 0.48 g/L, elevated IgM at 3.72 g/L, and normal IgG at 12.63 g/L. Complement levels showed elevated C3 at 1.53 g/L and normal C4 at 0.24 g/L. T cell subsets showed a total T lymphocyte percentage of 63.97%, helper T cell (CD3^+^ CD4^+^) percentage of 32.58%, cytotoxic T cell (CD3^+^ CD8^+^) percentage of 25.55%, and a CD4^+^/CD8^+^ ratio of 1.28, all within normal ranges. However, the total B lymphocyte percentage was reduced to 3.83% (reference range: 5.0%–18%). Cranial MRI showed cerebellar atrophy and cerebral white matter lesions in the left frontal lobe and bilateral parietal lobes. Representative cranial MRI of the affected sisters are shown in Figure 4. For the methods regarding whole exome sequencing, Sanger sequencing, and genetic analysis, please refer to the Supplementary Materials.

## Discussion

This study reports a novel mutation in the ATM gene, c.3062del (p.Val1021fs), identified in a Chinese family with two siblings affected by A-T. This mutation, which has not been previously documented in scientific literature, provides new insights into the genetic diversity of ATM-related diseases and contributes to the expanding catalog of ATM mutations associated with A-T (Kumada, 2019). The identification of this novel mutation underscores the importance of genetic testing in diagnosing A-T, particularly in populations where specific mutations may be underreported (Liu et al., 2016).

The ATM gene is associated with various mutation types, each leading to different clinical outcomes (Teive et al., 2015). To date, over 3,000 unique public DNA variants have been reported worldwide (https://databases.lovd.nl/shared/genes/ATM). These mutations primarily include missense, nonsense, frameshift, splice-site mutations, and large deletions (Huang et al., 2013). Two main forms of A-T have been reported, main category of more severe signs/symptoms categorized as classic or early-onset A-T and minority of cases have been referred to as mild or late-onset A-T (8). There is a well-established genotype-phenotype correlation in A-T, where the severity of the phenotype is influenced by the level of residual kinase activity determined by the specific genotype (Verhagen et al., 2009). In A-T, missense mutations which produce a mutant ATM protein with activity and leaky splice site mutations which allow expression of some normal ATM protein often lead to mild A-T, while nonsense, frameshift, and large genomic deletions are associated with classic A-T due to significant loss of protein function (Jacquemin et al., 2012; Schon et al., 2019; Taylor et al., 2015). Patients with classic A-T usually develop an ataxic gait in early childhood and become wheelchair dependency by adolescence (Petley et al., 2022). Patients with classic A-T are characterized by progressive cerebellar ataxia (65% onset before the age of 2) and is also accompanied by immunological complications (frequent infections, and an increased risk for malignancy) (Amirifar et al., 2020). Contrary to the classic A-T, the milder form does not present the cardinal features of A-T, such as ataxia, telangiectasia, and immunodeficiency. Reversely, its characteristics of movement disorders including chorea, myoclonic jerks, resting tremor, and dystonia are well recognized as the presenting manifestations of variant A-T (18). Recently, Liu et al. reports the identification of two novel missense mutations (p.I2683T and p. S2860P) in the ATM gene in a Chinese family. The study highlights that the proband exhibited dystonia without classical features of A-T, suggesting that ATM mutations can lead to varied clinical presentations, including isolated dystonia (Liu et al., 2023). The majority of ATM mutations causing A-T are frameshift and nonsense mutations, resulting in truncation of ATM protein (Huang et al., 2013; Micol et al., 2011; Amirifar et al., 2021). Huang et al. reported twelve novel ATM mutations identified in Chinese A-T patients, including four nonsense, five frameshift mutations (Huang et al., 2013). We reported a novel homozygous frameshift mutation, ATM_ex20 c.3062del (p.Val1021fs) that likely results in a truncated ATM protein, leading to loss of function (Taylor et al., 2015). In our case, the two affected sisters exhibited characteristics consistent with classic A-T (8).

The diagnosis of A-T is usually based on common clinical manifestations and laboratory tests that can be confirmed by genetic analysis (Amirifar et al., 2020). Telangiectasias do not often present in children lower than 5 years old and do not occur at all A-T patients. Moreover, a history of recurrent infections especially upper and lower respiratory tract infections, maybe another criterion to assist in the diagnosis (Perlman et al., 2003). Generally, delayed diagnosis in A-T patients is high because these patients are diagnosed when ataxia and oculocutaneous telangiectasia are both presents (Shao et al., 2023). Furthermore, delayed diagnosis is also associated with the lack of public awareness of the condition and the symptoms being initially subtle (Devaney et al., 2017). Moreover, as ultra-rare disease of A-T that many health professionals do not consider when assessing a child (Harari, 2016). As in our case, the parents had taken their child to see a doctor multiple times, but none of the physicians considered the possibility of this condition. It wasn't until the patient reached adulthood and visited our hospital’s Department of Eugenic Genetics that the possibility of this condition was considered, and a genetic diagnosis confirmed it. This case highlights the importance of increasing awareness of rare genetic diseases among both the public and healthcare professionals.

Immunodeficiency is a hallmark of A-T, mainly manifesting as low levels of immunoglobulins and recurrent infections, with IgA deficiency being particularly common (Takada et al., 2024; Pereira et al., 2024). Study showed that 60.8% of A-T patients exhibited IgA deficiency, while 28.6% present IgG deficiency. T- and B-lymphopenias were also frequently observed (Pereira et al., 2024). However, Shao et al. reported that 38.9% (7/18) of A-T patients in China did not exhibit immunodeficiency (Shao et al., 2023). In our study, both affected siblings displayed immunodeficiency, characterized by low IgA levels, slightly elevated IgM levels. Additionally, B-lymphopenias was observed in sister 2. Research has revealed that immunoglobulin deficiency in patients with A-T is attributed to the disrupted development of class-switched memory B cells. The deficiency of ATM gene impacts both the germinal center reaction and the selection of DNA repair pathways during class switching (Takada et al., 2024). Therefore, the observed decrease in IgA levels in our two patients may be related to the disrupted development of class-switched memory B cells. Moreover, both siblings had elevated AFP levels, an important biomarker for A-T (Renaud et al., 2020). AFP levels above 65 ng/mL have a specificity of 90%, a positive predictive value (PPV) of 83%, and a negative predictive value (NPV) of 73% for A-T diagnosis (Mariani et al., 2017). Schon et al. reported AFP levels in 57 A-T patients, with a mean serum AFP of 176 ng/mL (range: 2–600; standard deviation: 146) among 45 individuals. Only three (6.6%) had AFP levels within the normal range (Schon et al., 2019). ATM kinase plays multiple roles: it not only facilitates DNA repair, which explains the predisposition to cancers, but also influences transcription, potentially causing neurological symptoms and hepatic effects, leading to increased AFP(31). Collectively, the immunodeficiency manifestations—decreased IgA, reduced B lymphocytes, a slight increase in IgM, and significantly elevated AFP levels—support the diagnosis of A-T caused by novel frameshift mutations of the ATM gene in this Chinese family.

MRI of the brain is an essential tool for the diagnosis of A-T (33). Typically, MRI findings in A-T patients show cerebellar atrophy, cerebral white matter lesions, and enlarged fourth ventricles (Anheim et al., 2012; Sahama et al., 2014; Kose et al., 2024). While brain abnormalities in younger A-T patients are often confined to the cerebellum, older patients may exhibit more variable pathologies (Habek et al., 2008). In a study by Akturk et al., cranial MRI was performed on 66 out of 91 A-T patients, and pathological findings were observed in 47 cases (72.5%), including cerebellar atrophy (59.5%), cerebellar vermis hypoplasia (27.6%), and hyperintense signals in the cerebral white matter (12.7%) (Akturk et al., 2017). In our case, both affected sisters presented with cerebellar atrophy and cerebral white matter lesions (Figure 4). Unfortunately, due to delayed diagnosis, neither patient underwent cranial MRI during the early stages of the disease. For A-T patients, cranial MRI plays a crucial role by providing detailed brain imaging, enabling early detection, clear visualization of pathological changes, and effective monitoring of disease progression.

Besides cerebellar ataxia, neurologic features such as choreoathetosis, oculomotor apraxia, and resting tremor as well as neuromuscular disturbances also occur in A-T (Hiel et al., 2006). Verhagen et al., (2007) found that in typical A-T patients, with increasing age, especially in those over 8 years old, there is a gradual development of progressive axonal sensorimotor polyneuropathy, accompanied by increased muscle echo intensity on ultrasound, indicating fibrosis or fatty infiltration in the muscles. Moreover, Verhagen et al. conducted a neuropathological study of patients with classical and variant A-T. They discovered significant degenerative changes in the cerebellum, posterior columns of the spinal cord, and anterior horn neurons in classical A-T patients, which may be related to the muscle atrophy observed in these patients. In contrast, variant A-T patients exhibited milder neurodegenerative changes (Verhagen et al., 2012a). Pommerening et al. conducted a cohort study examining body composition, muscle strength, and hormonal status in patients with A-T. Their findings revealed that A-T patients had significantly lower fat-free mass, body cell mass, and manual muscle strength compared to healthy controls. The study underscores the prevalence of muscle wasting (myopenia) in A-T patients, which contributes to disease progression and fatigue. These results are particularly relevant to research on muscle atrophy in A-T, as they provide strong evidence of altered muscle mass and function in this patient population (Pommerening et al., 2015). Notably, in our case, both sisters exhibited classic symptoms of A-T. However, the older sister developed progressive muscle atrophy. In contrast, the younger sister showed no significant signs of muscle atrophy. Research has demonstrated that patients with A-T can exhibit significant clinical variability even when they carry similar mutations in the ATM gene (Amirifar et al., 2022). For example, in a Malian family with an ATM gene mutation, differences in age and symptom severity were observed. The older children, aged 14 and 10, exhibited more severe symptoms and had higher AFP levels compared to a 2-year-old sibling. Additionally, the older children presented with immunodeficiency and cerebellar atrophy, which were absent in the 2-year-old sibling (Landouré et al., 2013). Similarly, in a Chinese pedigree with A-T, a novel homozygous deletion mutation in the ATM gene was identified in three affected siblings. Despite sharing the same mutation, the siblings displayed differences in symptom onset, severity, and AFP levels, highlighting the complexity of phenotype manifestation in A-T (Chen et al., 2019). Our case also presented that patients with A-T carrying the same mutations exhibit significant clinical variability, indicating that other factors may contribute to these different clinical presentation, such as genetic alternations (Amirifar et al., 2022), epigenetic alteration including DNA methylation (McGrath-Morrow et al., 2020). However, the clear mechanism needs further investigation.

A-T patients have poor prognosis, and their survival time is approximately 25 years. The two most common causes of death in these patients are chronic pulmonary diseases and malignancy (Crawford et al., 2006). Regardless of which types of A-T, they are all associated with high risk for cancer (Verhagen et al., 2012b). Immunodeficiency and repeated exposure to pathogens and continuous stimulation with foreign antigens as another factor may be related to malignancy risk, especially IgA deficiency, which was significantly associated with the risk of lymphoid tumors (Suarez et al., 2015). However, patients with A-T’s life span and the quality of life could be prolonged by better management, such as immunoglobulin (IVIg) replacement therapy, antibiotic treatment, and prevention of unnecessary radiation exposure (Amirifar et al., 2021; Nowak-Wegrzyn et al., 2004). However, a recent study revealed that survival curves showed a mean survival time of 24.2 years, which was not affected by age of symptoms onset, age of diagnosis or time of diagnostic delay. Low IgG and male gender were significant risk factors associated with mortality (Shao et al., 2023). In our case, both affected sisters experienced recurrent respiratory infections during childhood. Tragically, the younger sister passed away at 30 years old due to a pulmonary infection and malignancy. Unfortunately, neither sister received an early diagnosis, which leaves it uncertain whether early intervention could have improved their prognoses.

Despite the significance of this finding, the study has several limitations. The small sample size, limited to a single family, restricts the generalizability of the results. Additionally, functional studies, including Western Blot analysis, were not performed to directly assess the impact of the c.3062delT mutation on ATM protein function. Further research is needed to confirm the pathogenicity of this mutation and to explore its frequency in broader populations. Moreover, while the clinical correlation in this case is strong, the variability in A-T presentation suggests that other genetic, epigenetic, or environmental factors may also influence disease severity and progression. Future studies should aim to investigate these factors to provide a more comprehensive understanding of A-T pathogenesis.

## Conclusion

In conclusion, this study identifies a novel ATM gene mutation, pathogenic frameshift mutation (ATM_ex20 c.3062delT, p. Val1021fs), in a Chinese family with A-T, expanding the known genetic diversity of this rare disorder. This finding has important implications for genetic diagnosis, counseling, and future research. As we continue to uncover the genetic basis of A-T, studies like this will play a crucial role in advancing our understanding of the disease and improving outcomes for affected individuals.

## Data Availability

The data presented in the study are deposited in the Sequence Read Archive repository (http://www.ncbi.nlm.nih.gov/bioproject/1182329), accession number PRJNA1182329.

## References

[B1] Aguado J. Gómez-Inclán C. Leeson H. C. Lavin M. F. Shiloh Y. Wolvetang E. J. (2022). The hallmarks of aging in Ataxia-Telangiectasia. Ageing Res. Rev. 79, 101653. 10.1016/j.arr.2022.101653 35644374

[B2] Akturk H. Sutcu M. Somer A. Piskin S. Acar M. Ozmen M. (2017). Ataxia telangiectasia in Turkey: multisystem involvement of 91 patients. World J. Pediatr. WJP 13 (13), 465–471. 10.1007/s12519-017-0011-z 28120234

[B3] Amirifar P. Mehrmohamadi M. Ranjouri M. R. Akrami S. M. Rezaei N. Saberi A. (2022). Genetic risk variants for class switching recombination defects in ataxia-telangiectasia patients. J. Clin. Immunol. 42, 72–84. 10.1007/s10875-021-01147-8 34628594 PMC8821084

[B4] Amirifar P. Ranjouri M. R. Lavin M. Abolhassani H. Yazdani R. Aghamohammadi A. (2020). Ataxia-telangiectasia: epidemiology, pathogenesis, clinical phenotype, diagnosis, prognosis and management. Expert Rev. Clin. Immunol. 16, 859–871. 10.1080/1744666X.2020.1810570 32791865

[B5] Amirifar P. Ranjouri M. R. Pashangzadeh S. Lavin M. Yazdani R. Moeini Shad T. (2021). The spectrum of ATM gene mutations in Iranian patients with ataxia-telangiectasia. Pediatr. Allergy Immunol. 32, 1316–1326. 10.1111/pai.13461 33547824

[B6] Amirifar P. Ranjouri M. R. Yazdani R. Abolhassani H. Aghamohammadi A. (2019). Ataxia-telangiectasia: a review of clinical features and molecular pathology. Pediatr. Allergy Immunol. 30, 277–288. 10.1111/pai.13020 30685876

[B7] Anheim M. Tranchant C. Koenig M. (2012). The autosomal recessive cerebellar ataxias. N. Engl. J. Med. 366, 636–646. 10.1056/NEJMra1006610 22335741

[B8] Cao J. Shen R. Zhang W. Mao B. Shi Q. Zhou R. (2019). Clinical diagnosis and genetic counseling of atypical ataxia-telangiectasia in a Chinese family. Mol. Med. Rep. 19, 3441–3448. 10.3892/mmr.2019.9992 30816533 PMC6471340

[B9] Chen W. Liu S. Hu H. Chen G. Zhu S. Jia B. (2019). Novel homozygous ataxia-telangiectasia (A-T) mutated gene mutation identified in a Chinese pedigree with A-T. Mol. Med. Rep. 20, 1655–1662. 10.3892/mmr.2019.10402 31257506 PMC6625389

[B10] Crawford T. O. Skolasky R. L. Fernandez R. Rosquist K. J. Lederman H. M. (2006). Survival probability in ataxia telangiectasia. Arch. Dis. Child. 91, 610–611. 10.1136/adc.2006.094268 16790721 PMC2082822

[B11] De Nardi L. Natale M. F. Messia V. Tomà P. De Benedetti F. Insalaco A. (2023). A child with polyarthritis and chronic lung disease: a case report of ataxia-telangiectasia. Ital. J. Pediatr. 49, 111. 10.1186/s13052-023-01509-5 37667293 PMC10478427

[B12] Devaney R. Pasalodos S. Suri M. Bush A. Bhatt J. M. (2017). Ataxia telangiectasia: presentation and diagnostic delay. Arch. Dis. Child. 102, 328–330. 10.1136/archdischild-2016-310477 27799156

[B13] Habek M. Brinar V. V. Rados M. Zadro I. Zarković K. (2008). Brain MRI abnormalities in ataxia-telangiectasia. Neurologist 14, 192–195. 10.1097/NRL.0b013e31815fa5a7 18469676

[B14] Harari S. (2016). Why we should care about ultra-rare disease. Eur. Respir. Rev. 25, 101–103. 10.1183/16000617.0017-2016 27246584 PMC9487247

[B15] Hiel J. A. P. van Engelen B. G. M. Weemaes C. M. R. Broeks A. Verrips A. ter Laak H. (2006). Distal spinal muscular atrophy as a major feature in adult-onset ataxia telangiectasia. Neurology 67, 346–349. 10.1212/01.wnl.0000224878.22821.23 16864838

[B16] Huang Y. Yang L. Wang J. Yang F. Xiao Y. Xia R. (2013). Twelve novel Atm mutations identified in Chinese ataxia telangiectasia patients. Neuromolecular Med. 15, 536–540. 10.1007/s12017-013-8240-3 23807571 PMC3732755

[B17] Jacquemin V. Rieunier G. Jacob S. Bellanger D. d'Enghien C. D. Laugé A. (2012). Underexpression and abnormal localization of ATM products in ataxia telangiectasia patients bearing ATM missense mutations. Eur. J. Hum. Genet. 20, 305–312. 10.1038/ejhg.2011.196 22071889 PMC3283185

[B18] Kose H. Karali Z. Bodur M. Cekic S. Kilic S. S. (2024). Neurological involvement in patients with primary immunodeficiency. Allergol. Immunopathol. Madr. 52, 85–92. 10.15586/aei.v52i1.961 38186198

[B19] Kumada S. (2019). Ataxia telangiectasia. Brain Nerve 71, 380–382. 10.11477/mf.1416201280 30988225

[B20] Landouré G. Mochel F. Meilleur K. Ly M. Sangaré M. Bocoum N. (2013). Novel mutation in the ATM gene in a Malian family with ataxia telangiectasia. J. Neurol. 260, 324–326. 10.1007/s00415-012-6738-5 23142947 PMC3566581

[B21] Lee J.-H. (2024). Oxidative stress and the multifaceted roles of ATM in maintaining cellular redox homeostasis. Redox Biol. 75, 103269. 10.1016/j.redox.2024.103269 39018798 PMC11301354

[B22] Liu X.-L. Wang T. Huang X.-J. Zhou H.-Y. Luan X.-H. Shen J.-Y. (2016). Novel ATM mutations with ataxia-telangiectasia. Neurosci. Lett. 611, 112–115. 10.1016/j.neulet.2015.11.036 26628246

[B23] Liu Z.-J. Wang Y.-L. Xu Y. (2023). Two novel heterozygote mutations of ATM in a Chinese family with dystonia-dominant ataxia telangiectasia and literature review. Front. Pediatr. 11, 975696. 10.3389/fped.2023.975696 37009283 PMC10050558

[B24] Mariani L. L. Rivaud-Péchoux S. Charles P. Ewenczyk C. Meneret A. Monga B. B. (2017). Comparing ataxias with oculomotor apraxia: a multimodal study of AOA1, AOA2 and AT focusing on video-oculography and alpha-fetoprotein. Sci. Rep. 7, 15284. 10.1038/s41598-017-15127-9 29127364 PMC5681651

[B25] McGrath-Morrow S. A. Ndeh R. Helmin K. A. Khuder B. Rothblum-Oviatt C. Collaco J. M. (2020). DNA methylation and gene expression signatures are associated with ataxia-telangiectasia phenotype. Sci. Rep. 10, 7479. 10.1038/s41598-020-64514-2 32366930 PMC7198504

[B26] Micol R. Ben Slama L. Suarez F. Le Mignot L. Beauté J. Mahlaoui N. (2011). Morbidity and mortality from ataxia-telangiectasia are associated with ATM genotype. J. Allergy Clin. Immunol. 128, 382–389.e1. 10.1016/j.jaci.2011.03.052 21665257

[B27] Mitiagin Y. Barzilai A. (2023). Ataxia-telangiectasia mutated plays an important role in cerebellar integrity and functionality. Neural Regen. Res. 18, 497–502. 10.4103/1673-5374.350194 36018153 PMC9727460

[B28] Nowak-Wegrzyn A. Crawford T. O. Winkelstein J. A. Carson K. A. Lederman H. M. (2004). Immunodeficiency and infections in ataxia-telangiectasia. J. Pediatr. 144, 505–511. 10.1016/j.jpeds.2003.12.046 15069401

[B29] Pereira R. A. Dantas E. O. Loekmanwidjaja J. Mazzucchelli J. T. L. Aranda C. S. Serrano M. E. G. (2024). Ataxia-telangiectasia in Latin America: clinical features, immunodeficiency, and mortality in a multicenter study. Immunol. Res. 72, 864–873. 10.1007/s12026-024-09494-5 38834764

[B30] Perez-Lloret S. van de Warrenburg B. Rossi M. Rodríguez-Blázquez C. Zesiewicz T. Saute J. A. M. (2021). Assessment of ataxia rating scales and cerebellar functional tests: critique and recommendations. Mov. Disord. 36, 283–297. 10.1002/mds.28313 33022077

[B31] Perlman S. Becker-Catania S. Gatti R. A. (2003). Ataxia-telangiectasia: diagnosis and treatment. Semin. Pediatr. Neurol. 10, 173–182. 10.1016/s1071-9091(03)00026-3 14653405

[B32] Petley E. Yule A. Alexander S. Ojha S. Whitehouse W. P. (2022). The natural history of ataxia-telangiectasia (A-T): a systematic review. PLoS One 17, e0264177. 10.1371/journal.pone.0264177 35290391 PMC9049793

[B33] Pommerening H. van Dullemen S. Kieslich M. Schubert R. Zielen S. Voss S. (2015). Body composition, muscle strength and hormonal status in patients with ataxia telangiectasia: a cohort study. Orphanet J. Rare Dis. 10, 155. 10.1186/s13023-015-0373-z 26645295 PMC4673730

[B34] Renaud M. Tranchant C. Koenig M. Anheim M. (2020). Autosomal recessive cerebellar ataxias with elevated alpha-fetoprotein: uncommon diseases, common biomarker. Mov. Disord. 35, 2139–2149. 10.1002/mds.28307 33044027

[B35] Rothblum-Oviatt C. Wright J. Lefton-Greif M. A. McGrath-Morrow S. A. Crawford T. O. Lederman H. M. (2016). Ataxia telangiectasia: a review. Orphanet J. Rare Dis. 11, 159. 10.1186/s13023-016-0543-7 27884168 PMC5123280

[B36] Sahama I. Sinclair K. Pannek K. Lavin M. Rose S. (2014). Radiological imaging in ataxia telangiectasia: a review. Cerebellum 13, 521–530. 10.1007/s12311-014-0557-4 24683014

[B37] Schmitz-Hübsch T. du Montcel S. T. Baliko L. Berciano J. Boesch S. Depondt C. (2006). Scale for the assessment and rating of ataxia: development of a new clinical scale. Neurology 66, 1717–1720. 10.1212/01.wnl.0000219042.60538.92 16769946

[B38] Schon K. van Os N. J. H. Oscroft N. Baxendale H. Scoffings D. Ray J. (2019). Genotype, extrapyramidal features, and severity of variant ataxia-telangiectasia. Ann. Neurol. 85, 170–180. 10.1002/ana.25394 30549301 PMC6590299

[B39] Shao L. Wang H. Xu J. Qi M. Yu Z. Zhang J. (2023). Ataxia-telangiectasia in China: a case report of a novel ATM variant and literature review. Front. Neurol. 14, 1228810. 10.3389/fneur.2023.1228810 37564729 PMC10411728

[B40] Shiloh Y. (1995). Ataxia-telangiectasia: closer to unraveling the mystery. Eur. J. Hum. Genet. 3, 116–138. 10.1159/000472285 7552141

[B41] Suarez F. Mahlaoui N. Canioni D. Andriamanga C. Dubois d'Enghien C. Brousse N. (2015). Incidence, presentation, and prognosis of malignancies in ataxia-telangiectasia: a report from the French national registry of primary immune deficiencies. J. Clin. Oncol. 33, 202–208. 10.1200/JCO.2014.56.5101 25488969

[B42] Takada S. Weitering T. J. van Os N. J. H. Du L. Pico-Knijnenburg I. Kuipers T. B. (2024). Causative mechanisms and clinical impact of immunoglobulin deficiencies in ataxia telangiectasia. J. Allergy Clin. Immunol. 153, 1392–1405. 10.1016/j.jaci.2023.12.029 38280573

[B43] Taylor A. M. R. Lam Z. Last J. I. Byrd P. J. (2015). Ataxia telangiectasia: more variation at clinical and cellular levels. Clin. Genet. 87, 199–208. 10.1111/cge.12453 25040471

[B44] Teive H. A. G. Moro A. Moscovich M. Arruda W. O. Munhoz R. P. Raskin S. (2015). Ataxia-telangiectasia - a historical review and a proposal for a new designation: ATM syndrome. J. Neurol. Sci. 355, 3–6. 10.1016/j.jns.2015.05.022 26050521 PMC5161405

[B45] Verhagen M. M. M. Abdo W. F. Willemsen M. A. A. P. Hogervorst F. B. L. Smeets D. F. C. M. Hiel J. A. P. (2009). Clinical spectrum of ataxia-telangiectasia in adulthood. Neurology 73 (73), 430–437. 10.1212/WNL.0b013e3181af33bd 19535770

[B46] Verhagen M. M. M. Last J. I. Hogervorst F. B. L. Smeets D. F. C. M. Roeleveld N. Verheijen F. (2012b). Presence of ATM protein and residual kinase activity correlates with the phenotype in ataxia-telangiectasia: a genotype-phenotype study. Hum. Mutat. 33, 561–571. 10.1002/humu.22016 22213089

[B47] Verhagen M. M. M. Martin J.-J. van Deuren M. Ceuterick-de Groote C. Weemaes C. M. R. Kremer B. H. P. H. (2012a). Neuropathology in classical and variant ataxia-telangiectasia. Neuropathology 32, 234–244. 10.1111/j.1440-1789.2011.01263.x 22017321

[B48] Verhagen M. M. M. van Alfen N. Pillen S. Weemaes C. M. R. Yntema J. L. Hiel J. A. P. (2007). Neuromuscular abnormalities in ataxia telangiectasia: a clinical, electrophysiological and muscle ultrasound study. Neuropediatrics 38, 117–121. 10.1055/s-2007-985899 17985259

