# Supplementary material for: Novel pathogenic ATM mutation with ataxia-telangiectasia in a Chinese family
Source: Front Genet. 2024 Nov 28;15:1491649. doi: 10.3389/fgene.2024.1491649 (PMC11638744; doi:10.3389/fgene.2024.1491649)
Supplement: Supplementary file 1 [file Table1.docx]

**Supplementary materials and methods**

**Whole exome sequencing**

DNA concentration was precisely quantified using the Qubit system. Samples with concentrations exceeding 0.5 µg were selected for library construction. The DNA libraries were constructed using a liquid-phase probe capture system, designed to efficiently enrich specific target regions of human DNA for high-throughput, high-depth sequencing on the Illumina platform. The capture experiment was conducted using a custom liquid-phase capture kit with biotin-labeled probes, following the manufacturer’s recommended reagents and consumables. The experimental procedure involved randomly fragmenting the genomic DNA into 150-200 bp fragments using a Covaris instrument. The fragmented DNA was then subjected to end-repair, A-tailing, and adapter ligation to prepare the sequencing library. Indexed libraries were pooled and hybridized with biotin-labeled probes in liquid phase. Magnetic beads coated with streptavidin were used to capture the exons of the targeted genes. Post-capture, the libraries were linearly amplified by PCR and subjected to quality control (QC). Libraries that passed QC were sequenced on the Illumina platform, with sequencing depth determined based on the effective concentration of the library and the desired data output. Based on the patients' clinical manifestations, particular attention was given to analyzing 1,170 genes associated with neurological diseases, including A2M, AAAS, AARS, ABAT, ABCB7, ATM, and others.

**Sanger sequencing**

Sanger sequencing was used to validate the candidate variants after data analysis. Forward and reverse primers were designed to amplify the fragments covering the variant sites. For each EDTA-anticoagulated whole blood sample, genomic total DNA was extracted using the nucleic acid extraction and purification kit (Xi'an Tianlong, China). The target fragments were amplified via PCR, followed by agarose gel electrophoresis of the PCR products. After confirming the quality and size of the bands, the target PCR fragments were purified and recovered. Purified PCR products were subjected to Sanger sequencing using the Applied Biosystems™ 3500xL Dx Genetic Analyzer, following the manufacturer’s protocol. Sequencing results were analyzed using SeqScape to locate the specific nucleotide position, and base changes or mutations were identified at the target sites.

**Genetic Analysis**

Data interpretation follows the guidelines of the American College of Medical Genetics and Genomics (ACMG)(1). Unless previously reported as pathogenic, data analysis will not focus on synonymous mutations, non-splice site intronic variants, or common benign polymorphisms. The report will primarily highlight mutations that are currently known or potentially associated with disease(2). All data interpretation is based on our current understanding of the relationship between diseases and pathogenic genes.

**References**

1 S. Richards, N. Aziz, S. Bale, D. Bick, S. Das, J. Gastier-Foster, et al., Standards and guidelines for the interpretation of sequence variants: a joint consensus recommendation of the American College of Medical Genetics and Genomics and the Association for Molecular Pathology. *Genet Med.* (2015) 17: 405-424.doi.org/10.1038/gim.2015.30

2 D.G. MacArthur, T.A. Manolio, D.P. Dimmock, H.L. Rehm, J. Shendure, G.R. Abecasis, et al., Guidelines for investigating causality of sequence variants in human disease. *Nature.* (2014) 508: 469-476.doi.org/10.1038/nature13127
